# Supplementary material for: SOD3 suppresses early cellular immune responses to parasite infection
Source: Nat Commun. 2024 Jun 8;15:4913. doi: 10.1038/s41467-024-49348-0 (PMC11162418; doi:10.1038/s41467-024-49348-0)
Supplement: Supplementary file 1 — Supplementary Information [file 41467_2024_49348_MOESM1_ESM.pdf]

Supplementary material

SOD3 suppresses early cellular immune responses to parasite infection

1. **Supplementary Method**
2. **Supplementary Figure 1: Impact of SOD3 knockout on parasite infection.**
3. **Supplementary Figure 2: Brain damage and inflammation were reduced in infected $SOD3^{-/-}$ mice.**
4. **Supplementary Figure 3: The proportion of IFN-γ-producing T cells significantly increased in $SOD3^{-/-}$ mice after parasite infection.**
5. **Supplementary Figure 4: IFN-γ signaling-related proteins were quantified by Western blotting in spleen at different time points post infection.**
6. **Supplementary Figure 5: SOD3 had minimal effect on the production of IL-10 and TNF-α in *P. berghei*-infected mice.**
7. **Supplementary Figure 6: SOD3 is a critical immune factor during parasite infection in both humans and mice.**
8. **Supplementary Figure 7: Uncropped Western blots.**
9. **Supplementary Figure 8：Gating strategy**
10. **Supplemental References**

Supplementary Method

Fluorescence-activated cell sorting (FACS) of macrophages and neutrophils

For isolation of splenic macrophages and neutrophils, spleens from WT mice were mechanically dissected through a 70-μm cell strainer at different time points after parasite infection. Macrophages were isolated with a BD FACSAria III by gating on $CD45^+$, $F4/80^+$, and $CD11b^+$ cells; neutrophils were isolated with a BD FACSAria III by gating on $CD45^+$, $F4/80^-$and $CD11b^+$ cells. The purity of sorted cells, as verified by FACS analysis, was more than 92%, which separated intact live cells from dead cells and enucleated cellular debris. The proportion of dead cells was determined by using a hemocytometer to count the cells stained with trypan blue and was lower than 5%.

Quantitative PCR with reverse transcription (RT–qPCR)

RNA from the above sorted cells was extracted using TRIzol solution (Invitrogen, CA, USA). Cat# 15596026) according to the manufacturer's instructions. RNA concentrations were determined using a NanoDrop2000 (Thermo Fisher Scientific). Reverse transcription reactions were performed using 5 μg of total RNA with HiScript III RT SuperMix for qPCR (+gDNA wiper) (Vazyme, Nanjing, China; Cat# R323).

RT–qPCR was performed with 5 ng of cDNA from sorted neutrophils and macrophages prior to infection and at different time points post-infection using a final concentration of 1 μM each for forward and reverse primers using SYBR qPCR Master Mix (Vazyme, Nanjing, China; Cat# Q711-00) on an Applied Biosystems 7500 fast Real-Time PCR System. All RT–qPCR amplicons were analyzed by agarose gel

electrophoresis to confirm the presence of a single specific band. Statistical analysis was performed using Applied Biosystems 7500 software. Target gene expression relative to that of an internal reference gene (*β-actin*) was calculated using the $2^{-\Delta\Delta Ct}$ method. Amplification primers were designed using PrimerBank (www.pga.mgh.harvard.edu/primerbank) and purchased from Sangon Biotech.

β-actin was amplified using the following primers:

forward primer, 5′-GGCTGTATTCCCCTCCATCG-3′

reverse primer, 5′-CCAGTTGGTAACAATGCCATGT-3′

SOD3 was amplified using the following primers:

forward primer, 5′- CCTTCTTGTTCTACGGCTTGC-3′

reverse primer, 5′-TCGCCTATCTTCTCAACCAGG-3′

Histopathological analysis

The spleens and brains from WT and SOD3$^{-/-}$ mice at different time points post-infection with *P. berghei* ANKA were fixed in 4% paraformaldehyde (Servicebio, China; cat. G1101-15ML) for 24 h at room temperature, embedded in paraffin, and sectioned at 5 μm. The spleen and brain sections were stained with hematoxylin and eosin, and the slides were scanned with a Panoramic SCAN and Panoramic Viewer (3D HISTECH).

Detection of cytokines

Sera were collected from WT and SOD3$^{-/-}$ mice at different time points post-infection, and the levels of IL-2, IL-10, MCP-1, IFN-γ, and TNF-α were determined using the

Mouse Cytokine Assay Kit according to the manufacturer's specifications (Integrated Biotech Solutions, IBS, Shanghai, China). A total of 1500 events were recorded for each preparation.

Western blotting

Protein from T cells or splenocytes sorted from WT and SOD3$^{-/-}$ mice prior to infection and at different time points post-infection were extracted with RIPA Lysis Buffer (50 mM Tris pH 7.4, 150 mM NaCl, 1% Triton X-100, 1% sodium deoxycholate, and 0.1% SDS, with a protease and phosphatase inhibitor cocktail for general use) (Biyuntian, China, Cat# P0013B), followed by denaturation with SDS–PAGE Protein Sample Loading Buffer (Biyuntian, Cat: P0286) at 100°C for 10 minutes. The total proteins were resolved by SDS–PAGE and transferred onto polyvinylidene difluoride (PVDF) membranes (Bio-Rad, Cat# 1620177). The membranes were blocked in 2% bovine serum albumin in PBS at 37°C for 60 min, incubated with primary antibodies overnight at 4 °C and then washed three times in 0.05% Tween-20 in PBS, followed by incubation with an HRP-conjugated goat anti-rabbit IgG secondary antibody (Biyuntian, Cat# A0208) or HRP-conjugated goat anti-mouse IgG secondary antibody (Biyuntian, Cat# A0216). Protein bands were quantified using ImageJ software. Target protein levels were normalized to that of β-actin. All WB experiments were repeated at least 3 times. Blots for phosphorylated p38 MAPK, phosphorylated JNK and phosphorylated AKT were stripped and reprobed for total p38 MAPK, total JNK and total AKT, respectively.

A detailed list of primary antibodies including pan-AKT1/2/3 Antibody (Affinity, AF6261), P-AKT(Affinity, AF3262), JNK1+JNK2+JNK3 Rabbit Monoclonal Antibody (Biyuntian Biotechnology Institute, AF1048 and Affinity, AF6318), Phospho-JNK1/JNK2/JNK3 (Thr183/Thr183/Thr221) Rabbit Monoclonal Antibody (Biyuntian Biotechnology Institute, AF1762 and Affinity, AF3318), p38 MAPK Ab (Affinity, AF6456 and Biyuntian Biotechnology Institute,AF7668), Phospho-p38 MAPK Mouse Monoclonal Antibody (Biyuntian Biotechnology Institute, AM063-1), SIRT1 (Jingjie PTM Biolabs, PTM5021), STAT5a Rabbit Monoclonal Antibody (Biyuntian Biotechnology Institute, AF2038) , STAT5b Rabbit Monoclonal Antibody (Biyuntian Biotechnology Institute, AG3329) , TLR1/Toll-like Receptor 1 Rabbit Polyclonal Antibody (Biyuntian Biotechnology Institute AF8178) ,TLR4 (Biyuntian Biotechnology Institute, AF8187) ,TLR7 (Biyuntian Biotechnology Institute, AF0300) ,TLR8 (Biyuntian Biotechnology Institute AF8190),TLR9 (Biyuntian Biotechnology Institute, AF8193) , NF-kB p65 Rabbit Monoclonal Antibody (Biyuntian Biotechnology Institute, AF1234) , Phospho-NF-κB p65 (Ser276) Rabbit Polyclonal Antibody (Biyuntian Biotechnology Institute, AF5875 ,Biyuntian Biotechnology Institute AF5878, Biyuntian Biotechnology Institute, AF5881) , ERK1 Rabbit Monoclonal Antibody or ERK1/2 Rabbit Monoclonal Antibody (Biyuntian Biotechnology Institute, AF1315, Biyuntian Biotechnology Institute, AF1051), Phospho-ERK1-Thr202/Tyr204+ERK2-Thr185/Tyr187 Rabbit Polyclonal Antibody (Biyuntian Biotechnology Institute, AF5818), β-Actin Rabbit Monoclonal Antibody (Biyuntian Biotechnology Institute, AF5003 and Affinity, BP001M and Servicebio,

GB12001).

Flow cytometry analysis of immune cells

Flow cytometry was performed using a BD FACSAria III. Spleens from SOD3$^{-/-}$ and WT mice at different time points after parasite infection were prepared for dissection as described in the section 'Fluorescence-activated cell sorting'. Erythrocytes in the blood and splenic single-cell suspensions were lysed in lysis buffer for 10 min, washed in 10 mL of PBS, incubated with a purified anti-mouse CD16/32 antibody, fixed with a freshly prepared True-NuclearTM Transcription Factor Buffer Set (BioLegend, USA, Cat# 424401), washed twice with True-NuclearTM Perm Buffer (BioLegend), and incubated with specific antibodies or isotype controls according to the manufacturer's guidelines. The specific antibodies included anti-CD3 (BioLegend, clone 17A2), anti-CD8 (Tonbo, China, Cat# 50-0081), anti-CD4 (BioLegend, clone GK1.5), anti-IFN-γ (BioLegend, clone XMG1.2), anti-NK1.1 (BioLegend, clone S17016D), anti-IL-2 (BioLegend, clone JES6-5H4), and anti-CD45 (BioLegend, clone 30-F11) antibodies.

T cell isolation

Isolation of T cells were purified from splenocytes using Mouse CD4^{+} T Cell Isolation Kit (Negative Selection, M7401M, UElandy Inc, China) or Mouse CD8^{+} T Cell Isolation Kit (Negative Selection, M7402S, UElandy Inc, China) followed by magnetic cell sorting. The purity of T cell populations enriched by sorting was ≥95%.

T cell culture

Mouse T cells were isolated from spleen 4–6 weeks old wild-type C57BL6. Cells was separated conventionally, and centrifugated and washed by PBS to prepare lymphocyte suspension. T cells were then activated with combination of 5 μg/mL of mouse anti-CD3 (Catalog No. 102115, BioLegend) and 5 μg/mL of anti-CD28 (Catalog No. 100339, BioLegend) for 24 h in RPMI-1640 medium, GlutaPlus, HEPES, sodium pyruvate (G4535-500ML; Servicebio, Wuhan, China) supplemented with 10% fetal bovine serum, 100 U/mL penicillin, and 100 μg/mL streptomycin (servicebio, G4003-100ml) and recombinant mouse IL-2 (carrier-free, 300U/ml, BioLegend, USA, 575402) at 37°C in an incubator.

Cell binding assay with recombinant SOD3

His-SOD3 fusion protein was incubated with spleen immune cells as previously described[1]. The SOD3 bound to the cell surface was detected by flow cytometry using anti-SOD3- Rabbit antibody (Affinity, DF7753) and Alexa Fluor™ 488-conjugated goat anti-Rabbit IgG (H+L) Highly Cross-Adsorbed Secondary Antibody (Invitrogen, A-11034). For the control staining, cells were treated with a non-specific rabbit IgG as the primary antibody, using the same AF488-conjugated goat anti-rabbit IgG as the secondary antibody. Flow cytometric analyses were performed using EV450 -conjugated anti-mouse CD45 (Elabscience, E-AB-F1136Q), Percp cy5.5-conjugated anti-mouse CD4 (Biolegend, 100433), APC-conjugated anti-mouse CD8a (Biolegend,

100711), PE-conjugated anti-mouse NK1.1 (Elabscience, E-AB-F0987D).

Cell activation analysis

Isolated T cells were activated with combinations of 5 μg/mL of mouse anti-CD3 and cultivated in RPMI-1640 with 5 μg/mL of anti-CD28 and 300U/mL IL-2 for 24 h. Then, the cells in the SOD3-treated group were treated with rSOD3 (100 U/mL) for 24 hours, while the control group was treated with the same dose of PBS. After 48 h of cell culture, T cells were then harvested, washed, and stained with EV450 -conjugated anti-mouse CD45 (Elabscience, E-AB-F1136Q), APC-conjugated anti-mouse CD25 (Biolegend, 102012), FITC-conjugated anti-mouse CD8 antibodies for analyzed by flow cytometry.

T cell differentiation analysis

Isolated T cells were activated with combinations of 5 μg/mL of mouse anti-CD3 and cultivated in RPMI-1640 with 5 μg/mL of anti-CD28 and 300U/mL IL-2 for 24 h. Then, the cells in the SOD3-treated group were treated with rSOD3 (100 U/mL) for 24 hours, while the control group was treated with the same dose of PBS. Cells were then harvested, washed and stained with EV450 -conjugated anti-mouse CD45 (Elabscience, E-AB-F1136Q), FITC-conjugated anti-mouse CD8 (Biolegend, 100706), BV605-conjugated anti-mouse CD44 (Biolegend, 103047), PE/Cy7-conjugated anti-mouse CD62L (Biolegend, 104417) antibodies for analyzed by flow cytometry.

IL-2 and IFN-γ production analysis

T cells were cultured as before and incubated with rSOD3 (100 U/mL) or SP600125 (ATP-competitive JNK inhibitor, Selleck, S1460) for 24 h. Cells were then harvested, washed and stained with antibodies for analyzed by flow cytometry. BV605-conjugated anti-mouse IL-2, APC-conjugated anti-mouse IFN-γ, Pacific Blue™-conjugated anti-mouse CD45, FITC-conjugated anti-mouse CD8 was purchased from Biolegend.

Mass cytometry analysis of Th1 cells in *P. y. yoelii*-infected mice

Mass cytometry (CyTOF) was performed as previously described[2]. Spleens from WT mice and SOD3$^{-/-}$ mice were mechanically dissected through a 70-µm cell strainer at different time points after *P. y. yoelii* infection. The single cells were first stained with cisplatin viability dye, and then stained with 144Nd labeling IL-2, 165Ho-labeling IFN-γ, 147Sm-labeling CD45, 168Er-labeling CD8. All antibodies were purchased from polarisbiology (China, Jiangsu).

Supplemental Figures

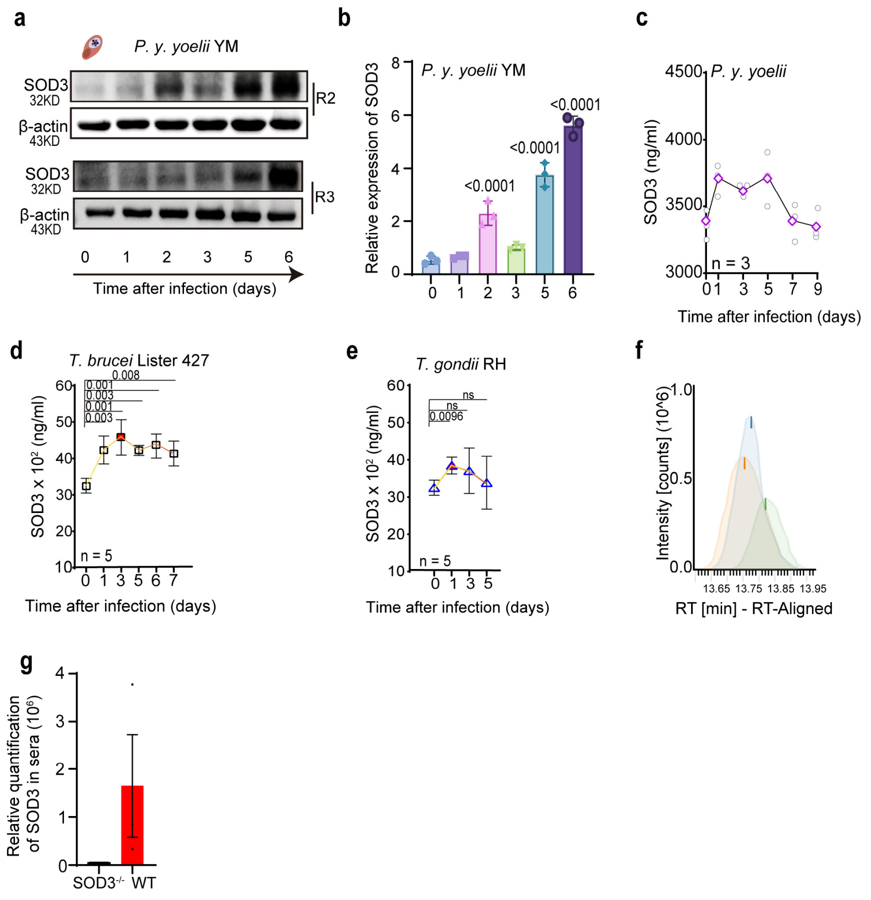

Supplementary Figure 1. Impact of SOD3 knockout on parasite infection. (a and **b)** SOD3 expression was determined in WT mice at different time points post-infection with *P. y. yoelii* YM. Two other independent repeats of Figure 1a are shown. **(c)** SOD3 levels in sera were elevated after infection with *P. y. yoelii* at different time points post-infection, with peak effects observed at 3 days and 7 days post-infection. **(d)** Serum SOD3 expression was elevated after infection with *T. b. brucei*, with peaks observed at 3 days post-infection, respectively. To test the levels of SOD3 in WT mice infected with *T. b. brucei*, serum was collected from mice on days 1, 3, 5, 6 and 7 post-challenge *T. b. brucei*. Average survival time of WT mice was 8 days from the time of challenge with *T. b. brucei*. **(e)** Serum SOD3 expression was elevated after infection with *T. gondii* RH, with peaks observed at one day post-infection, respectively. To test the levels of SOD3 in WT mice,

serum was collected from mice on alternate days from day 1 to 5 post-infection of *T. gondii* RH. Average survival time of WT mice was 7 days from the time of challenge with *T. gondii* RH. Due to the death of these mice after *T. gondii* RH infection, mouse serum samples could not be collected at days 7 post -infection. **(f** and **g)** Serum SOD3 levels were quantified using an Orbitrap Exploris 480 mass spectrometer for both SOD3$^{-/-}$ mice and WT mice after *P. berghei* ANKA infection. SOD3$^{-/-}$ mice completely lack SOD3 expression. Source data are provided as a Source Data file.

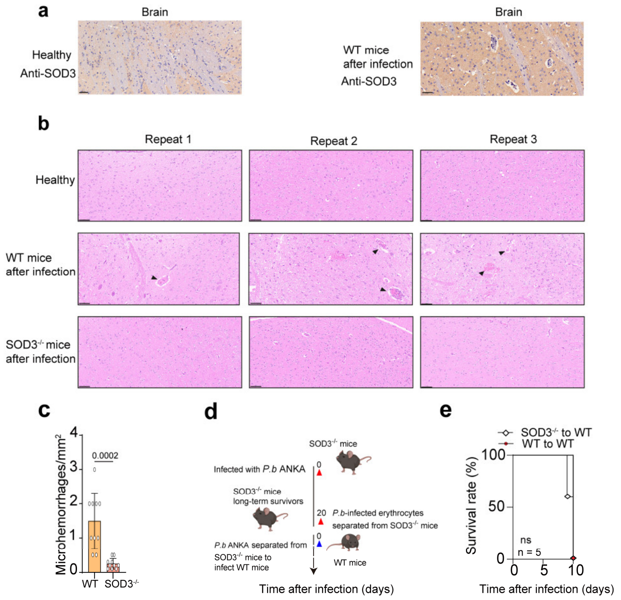

Supplementary Figure 2. Brain damage and inflammation were reduced in infected SOD3$^{-/-}$ mice. (a) Representative images of brain tissues from healthy and *P. berghei* ANKA-infected mice immunohistochemically stained for SOD3. **(b)** Representative H&E staining of the cerebral cortex at 63× magnification. Scale bars = 50 µm. **(c)** Statistical evaluation of microvessel density (MVD). An increase in MVD was observed in WT mice after *P. berghei* ANKA infection. Means ± SEMs, unpaired *t* test. **(d)** Schematic representation of *P. berghei* ANKA isolation from both SOD3$^{-/-}$ mice and WT mice. The mice illustration was created with http://BioRender.com (publishing license: XZ26UIL8LK). **(e)** *P. berghei* isolated from SOD3$^{-/-}$ mice and WT mice displayed similar infectivity in naïve micen = 5. Kaplan–Meier survival curves were calculated using the survival time for each mouse in all groups, and significance was determined by the log-rank test. Source data are provided as a Source Data file.

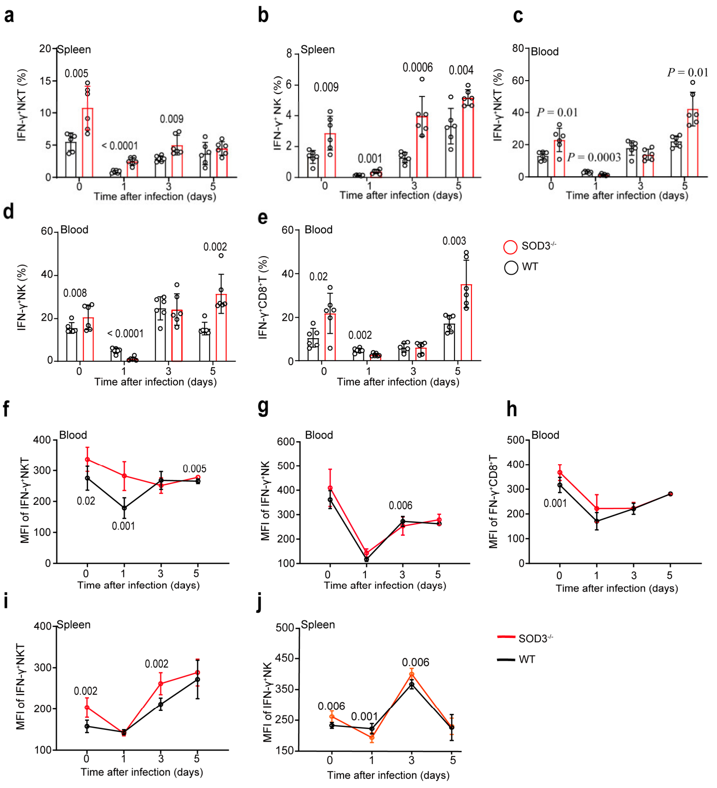

Supplementary Figure 3. The proportion of IFN-γ-producing T cells significantly increased in SOD3$^{-/-}$ mice after parasite infection. (a and **b)** The proportions of IFN-γ^{+} NKT cells and IFN-γ^{+} NK cells were quantified in both WT and SOD3$^{-/-}$ mice (n = 6) at different time points after infection with *P. berghei*. **(c-e)**, Quantification of the frequencies of splenic and blood IFN-γ^{+} NKT cells, IFN-γ^{+} NK cells and IFN-γ^{+} CD8^{+} T cells in both WT and SOD3$^{-/-}$ mice (n = 6) at different time points after infection with *P. berghei* is shown. **(f-**H**)** The relative mean fluorescence intensity (MFI) of IFN-γ in blood NKT cells, NK cells and NK cells was determined. **(i** and **j)** The MFI of IFN-γ in splenic NKT cells and NK cells is also shown. Student's *t* test was used to test for significant differences between two groups. Source data are provided as a Source Data file.

a

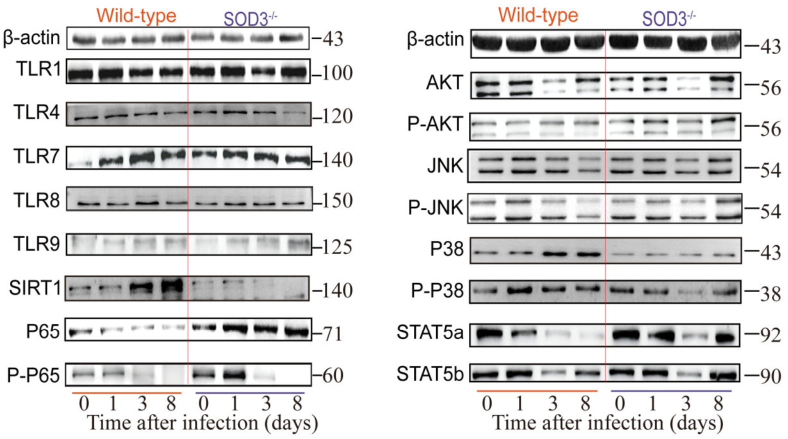

Supplementary Figure 4. IFN-γ signaling-related proteins were quantified by Western blotting in spleen at different time points post infection. **(a)** Representative immunoblots using antibodies against β-actin, phospho-AKT, AKT, phospho-JNK, JNK, phospho-p38, p38, TLR1, TLR4, TLR7, TLR8, TLR9, SIRT1, P65, P-P65, STAT5a and STAT5b are shown.

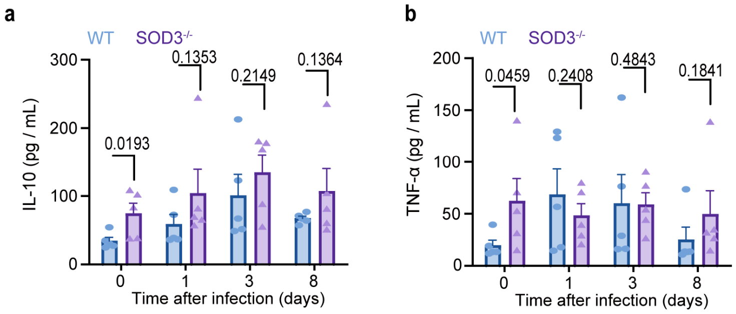

Supplementary Figure 5. SOD3 had minimal effect on the production of IL-10 and TNF-α in *P. berghei*-infected mice. (a and b) The levels of IL-10 and TNF-α in the sera were detected in both WT and $SOD3^{-/-}$ mice at different time points after *P. berghei* infection.

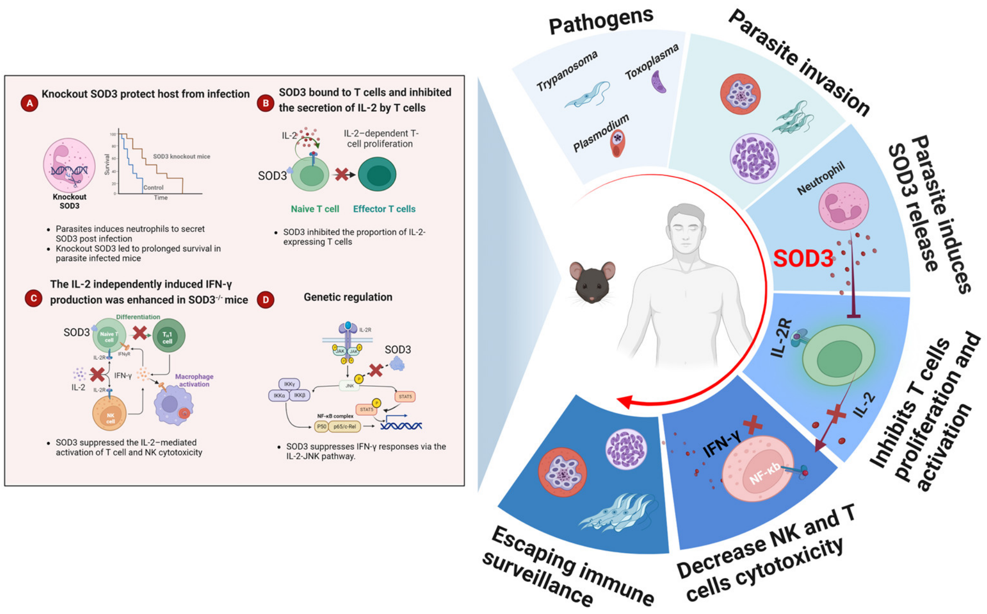

Supplementary Figure 6. SOD3 is a critical immune factor during parasite infection in both humans and mice. SOD3 secreted by neutrophils efficiently suppressed IL-2 production by T cells and consequently reduced the recruitment and responses of IFN-γ^{+} T cells and NK cells to invading parasites. The illustration was created with http://BioRender.com (publishing license: UW26UICE18).

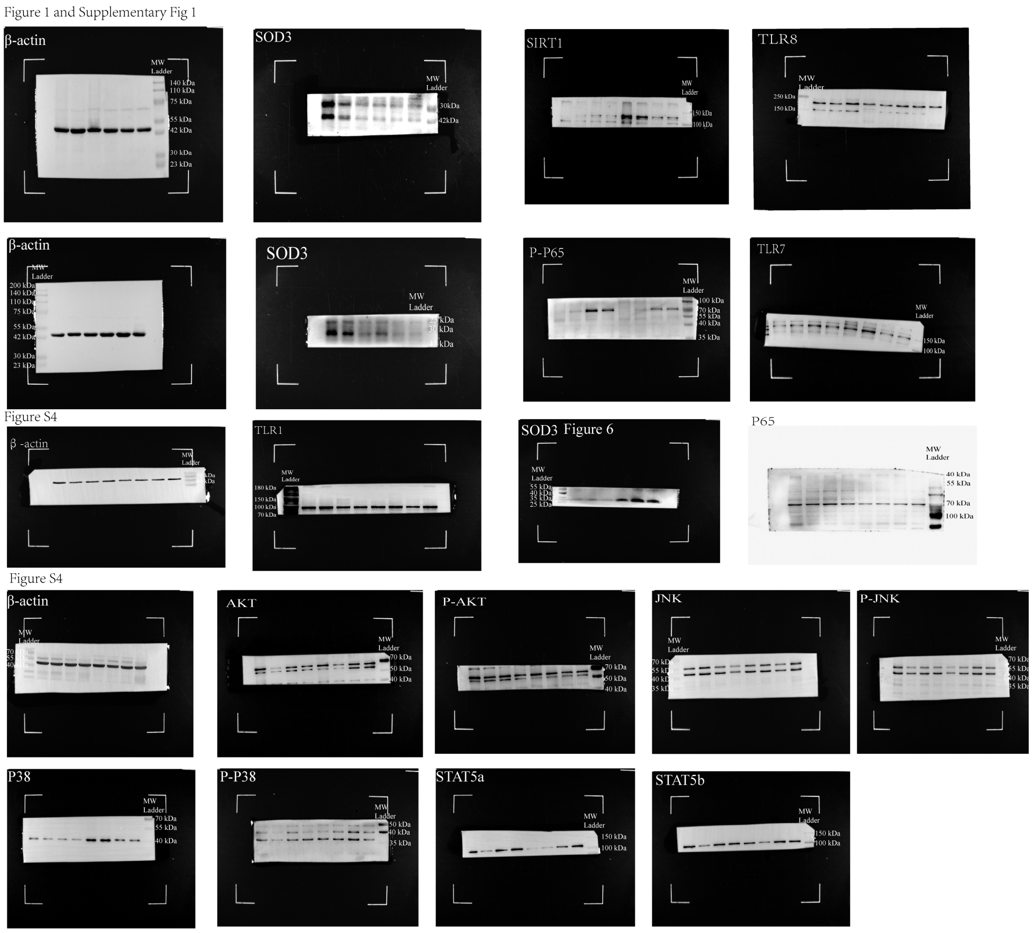

Supplementary Figure 7. Uncropped Western blots.

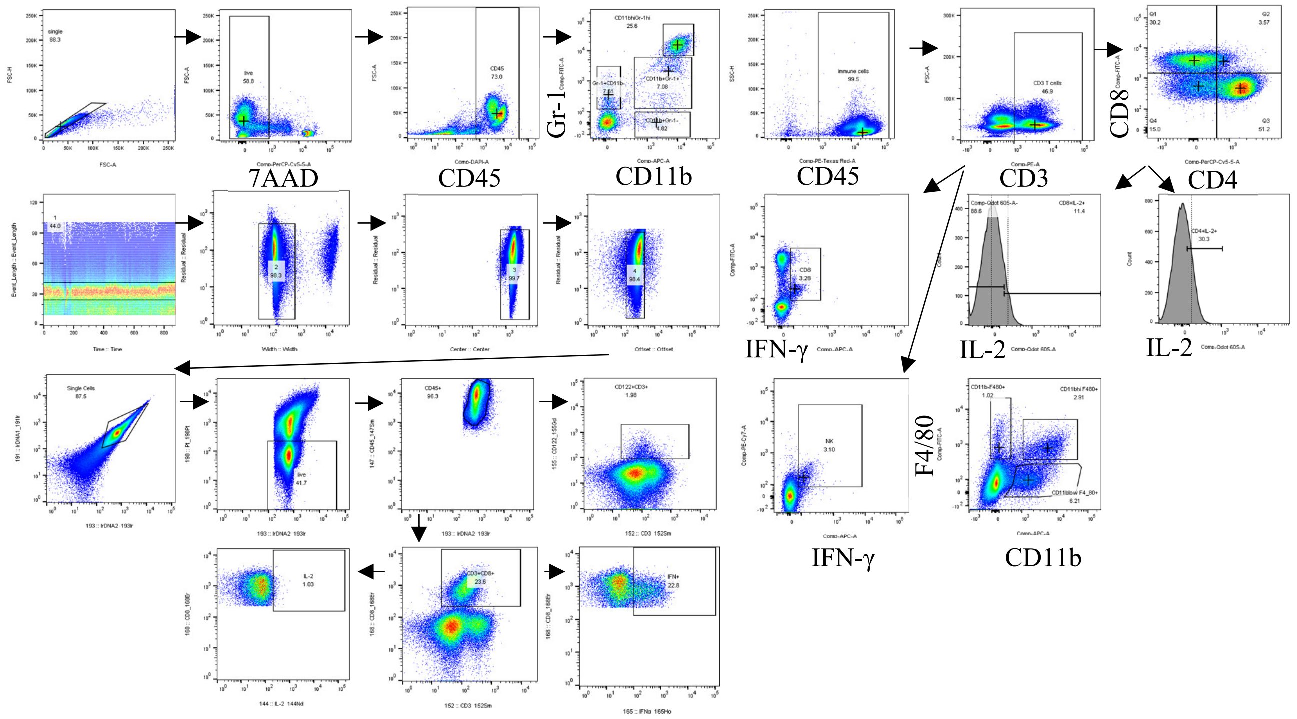

Supplementary Figure 8. Gating strategy

Supplemental References

1. Hu, Lili et al. "The dynamic uptake and release of SOD3 from intracellular stores in macrophages modulates the inflammatory response." *Redox biol* **26**, 101268 (2019).
2. Leipold MD, Newell EW & Maecker HT Multiparameter phenotyping of human PBMCs using mass cytometry. *Methods Mol. Biol* **1343**, 81–95 (2015)
