# Supplementary material for: SOD3 suppresses early cellular immune responses to parasite infection
Source: Nat Commun. 2024 Jun 8;15:4913. doi: 10.1038/s41467-024-49348-0 (PMC11162418; doi:10.1038/s41467-024-49348-0)
Supplement: Supplementary file 3 — Reporting Summary [file 41467_2024_49348_MOESM3_ESM.pdf]

nature portfolio

Corresponding author(s): Qijun Chen

Last updated by author(s): May 10, 2024

Reporting Summary

Nature Portfolio wishes to improve the reproducibility of the work that we publish. This form provides structure for consistency and transparency in reporting. For further information on Nature Portfolio policies, see our Editorial Policies and the Editorial Policy Checklist.

Statistics

For all statistical analyses, confirm that the following items are present in the figure legend, table legend, main text, or Methods section.

n/a	Confirmed	
☐	☒	The exact sample size (*n*) for each experimental group/condition, given as a discrete number and unit of measurement
☐	☒	A statement on whether measurements were taken from distinct samples or whether the same sample was measured repeatedly
☐	☒	The statistical test(s) used AND whether they are one- or two-sided<br>*Only common tests should be described solely by name; describe more complex techniques in the Methods section.*
☒	☐	A description of all covariates tested
☐	☒	A description of any assumptions or corrections, such as tests of normality and adjustment for multiple comparisons
☐	☒	A full description of the statistical parameters including central tendency (e.g. means) or other basic estimates (e.g. regression coefficient) AND variation (e.g. standard deviation) or associated estimates of uncertainty (e.g. confidence intervals)
☐	☒	For null hypothesis testing, the test statistic (e.g. *F*, *t*, *r*) with confidence intervals, effect sizes, degrees of freedom and *P* value noted<br>*Give P values as exact values whenever suitable.*
☒	☐	For Bayesian analysis, information on the choice of priors and Markov chain Monte Carlo settings
☒	☐	For hierarchical and complex designs, identification of the appropriate level for tests and full reporting of outcomes
☐	☒	Estimates of effect sizes (e.g. Cohen's *d*, Pearson's *r*), indicating how they were calculated

Our web collection on statistics for biologists contains articles on many of the points above.

Software and code

Policy information about availability of computer code

Data collection	AniView600 multimode in vivo animal imaging system (Guangzhou Biolight Biotechnology Co., Ltd., China) was used to detect Pb. ANKA-luc in mice, and software was use to analyze data.The SOD3 protein was detect by use LC–MS/MS (Orbitrap Exploris 480). Cells was analysis by BD FACSAria III.
Data analysis	Proteome Discoverer 2.4 (Thermo), Applied Biosystems 7500 software,Panoramic SCAN and Panoramic Viewer (3D HISTECH), R (version 4.0.3), Graph Pad Prism (version 9).

For manuscripts utilizing custom algorithms or software that are central to the research but not yet described in published literature, software must be made available to editors and reviewers. We strongly encourage code deposition in a community repository (e.g. GitHub). See the Nature Portfolio guidelines for submitting code & software for further information.

Data

Policy information about availability of data

All manuscripts must include a data availability statement. This statement should provide the following information, where applicable:

- Accession codes, unique identifiers, or web links for publicly available datasets
- A description of any restrictions on data availability
- For clinical datasets or third party data, please ensure that the statement adheres to our policy

All data supporting the findings of this study are available within the paper. Source data are provided with this paper.

Research involving human participants, their data, or biological material

Policy information about studies with human participants or human data. See also policy information about sex, gender (identity/presentation), and sexual orientation and race, ethnicity and racism.

Reporting on sex and gender	Gender of human donors was provided in the method, and was not considered in the study design.
Reporting on race, ethnicity, or other socially relevant groupings	No reporting of race, ethnicity or other socially relevant groupings was made in this report.
Population characteristics	P. falciparum malaria patients, with the ages range from 21 to 70.
Recruitment	No selection bias present in this study to impact results.
Ethics oversight	This study was approved by the Ethical Committee of the Chinese Academy of Medical Sciences (approval no. IPB-2016-2) and Institutional Review Board (no. PRAMS0034319) of the Pennsylvania State University.

Note that full information on the approval of the study protocol must also be provided in the manuscript.

Field-specific reporting

Please select the one below that is the best fit for your research. If you are not sure, read the appropriate sections before making your selection.

☒ Life sciences ☐ Behavioural & social sciences ☐ Ecological, evolutionary & environmental sciences

For a reference copy of the document with all sections, see nature.com/documents/nr-reporting-summary-flat.pdf

Life sciences study design

All studies must disclose on these points even when the disclosure is negative.

Sample size	No sample size calculation was performed.The sample size was determined based on the availability of samples to provide at least 3 biological replicate for a sufficient statistical power.
Data exclusions	No data were excluded.
Replication	All experiments were performed in triplicates and fully replicated in all instances.
Randomization	All mice were assigned randomly.
Blinding	Investigators were not blinded during sample collection or analysis. The process of sample collection is the same for all samples, and no differences between samples are apparent utile sample imaging.Due to the automated nature of sample analysis, blinding was not required,as all samples were analyzed identically.

Reporting for specific materials, systems and methods

We require information from authors about some types of materials, experimental systems and methods used in many studies. Here, indicate whether each material, system or method listed is relevant to your study. If you are not sure if a list item applies to your research, read the appropriate section before selecting a response.

Materials & experimental systems

n/a	Involved in the study	
☐	☒	Antibodies
☒	☐	Eukaryotic cell lines
☒	☐	Palaeontology and archaeology
☐	☒	Animals and other organisms
☒	☐	Clinical data
☒	☐	Dual use research of concern
☒	☐	Plants

Methods

n/a	Involved in the study	
☒	☐	ChIP-seq
☐	☒	Flow cytometry
☒	☐	MRI-based neuroimaging

Antibodies

Antibodies used	A detailed list of primary antibodies including pan-AKT1/2/3 Antibody (Affinity, AF6261), P-AKT(Affinity, AF3262), JNK1+JNK2

Antibodies used

+JNK3Rabbit Monoclonal Antibody (Biyuntian Biotechnology Institute, AF1048 and Affinity, AF6318), Phospho-JNK1/JNK2/JNK3 (Thr183/
Thr183/Thr221) Rabbit Monoclonal Antibody (Biyuntian Biotechnology Institute, AF1762 and Affinity, AF3318), p38 MAPK Ab (Affinity, AF6456 and Biyuntian Biotechnology Institute,AF7668), Phospho-p38 MAPK Mouse Monoclonal Antibody (Biyuntian Biotechnology Institute, AM063-1), SIRT1 (Jingjie PTM Biolabs, PTM5021), STAT5a Rabbit Monoclonal Antibody (Biyuntian Biotechnology Institute, AF2038) , STAT5b Rabbit Monoclonal Antibody (Biyuntian Biotechnology Institute, AG3329) , TLR1/Toll-like Receptor 1 Rabbit Polyclonal Antibody (Biyuntian Biotechnology Institute AF8178) ,TLR4 (Biyuntian Biotechnology Institute, AF8187) ,TLR7 (Biyuntian Biotechnology Institute, AF0300) ,TLR8 (Biyuntian Biotechnology Institute AF8190),TLR9 (Biyuntian Biotechnology Institute, AF8193) , NF-kB p65 Rabbit Monoclonal Antibody (Biyuntian Biotechnology Institute, AF1234) , Phospho-NF-κB p65 (Ser276) Rabbit Polyclonal Antibody (Biyuntian Biotechnology Institute, AF5875 ,Biyuntian Biotechnology Institute AF5878, Biyuntian Biotechnology Institute, AF5881) , ERK1 Rabbit Monoclonal Antibody or ERK1/2 Rabbit Monoclonal Antibody (Biyuntian Biotechnology Institute, AF1315, Biyuntian Biotechnology Institute, AF1051), Phospho-ERK1-Thr202/Tyr204+ERK2-Thr185/Tyr187 Rabbit Polyclonal Antibody (Biyuntian Biotechnology Institute, AF5818), β-Actin Rabbit Monoclonal Antibody (Biyuntian Biotechnology Institute, AF5003 and Affinity, BP001M and Servicebio, GB12001).

Validation

The data have provided in the manuscript.All primary antibodies were used at a 1:1,000 dilution, and secondary antibody was used at 1:2,000.

Animals and other research organisms

Policy information about studies involving animals; ARRIVE guidelines recommended for reporting animal research, and Sex and Gender in Research

Laboratory animals

Six to eight-week old female and male C57BL/6 mice were obtained from Liaoning Changsheng Biological Technology Company (Liaoning, China).
Six to eight-week old female and male C57BL/6-Sod3tm1cyagen (SOD3-/-) mouse strains (Serial Number: KOCMP-22050-Sod3) were purchased from Cyagen Biosciences (Suzhou, China). C57BL/6-Sod3tm1cyagen mice were maintained on the C57BL/6 genetic background.

Wild animals

This study did not involve wild animals.

Reporting on sex

This study does not involve gender studies. Female and male mice had the same results.

Field-collected samples

This study did not involve samples collected from the field.

Ethics oversight

The animal experiments were conducted according to the animal husbandry guidelines of Shenyang Agricultural University (permit no. SYXK<Liao>2021-0010).

Note that full information on the approval of the study protocol must also be provided in the manuscript.

Plants

Seed stocks

Report on the source of all seed stocks or other plant material used. If applicable, state the seed stock centre and catalogue number. If plant specimens were collected from the field, describe the collection location, date and sampling procedures.

Novel plant genotypes

Describe the methods by which all novel plant genotypes were produced. This includes those generated by transgenic approaches, gene editing, chemical/radiation-based mutagenesis and hybridization. For transgenic lines, describe the transformation method, the number of independent lines analyzed and the generation upon which experiments were performed. For gene-edited lines, describe the editor used, the endogenous sequence targeted for editing, the targeting guide RNA sequence (if applicable) and how the editor was applied.

Authentication

Describe any authentication procedures for each seed stock used or novel genotype generated. Describe any experiments used to assess the effect of a mutation and, where applicable, how potential secondary effects (e.g. second site T-DNA insertions, mosiacism, off-target gene editing) were examined.

Flow Cytometry

Plots

Confirm that:

☒ The axis labels state the marker and fluorochrome used (e.g. CD4-FITC).

☒ The axis scales are clearly visible. Include numbers along axes only for bottom left plot of group (a 'group' is an analysis of identical markers).

☒ All plots are contour plots with outliers or pseudocolor plots.

☒ A numerical value for number of cells or percentage (with statistics) is provided.

Methodology

Sample preparation

Samples were collected and then red blood cells were lysed. Following pre-incubation with a purified anti-mouse CD16/32

Sample preparation	antibody, the cells were then incubated with specific antibodies or isotype controls, according to the manufacturer's guidelines.
Instrument	Fluorescence-activated cell sorting Aria III flow cytometer (BD Biosciences, San Jose, CA, USA)
Software	Flow data was collected and analyzed using the flow software BD FACS Diva.
Cell population abundance	Aliquots of sorted cells were reanalyzed to ensure purity, which was greater than 90%.
Gating strategy	The gating strategy was defined as compared to non-stained cells. Non-specific rabbit IgG was used as the primary antibody for control samples in SOD3 binding experiment.

☒ Tick this box to confirm that a figure exemplifying the gating strategy is provided in the Supplementary Information.
